# Supplementary material for: An open-source framework for large-scale, flexible evaluation of biomedical text mining systems
Source: J Biomed Discov Collab. 2008 Jan 29;3:1. doi: 10.1186/1747-5333-3-1 (PMC2276192; doi:10.1186/1747-5333-3-1)
Supplement: Additional file 1 — Evaluation platform source code. The additional file contains the software evaluation framework discussed in this paper. The most current release can be downloaded from SourceForge.net [1]. See the accompanying README file for instructions on installation and use. Also included in the distribution is a collection of UIMA wrappers for some commonly used BioNLP tools and annotated corpora. [file 1747-5333-3-1-S1.zip › bionlp_uima_repository-1.0.1/docs/api/src-html/edu/uchsc/ccp/util/nlp/mention/comparison/IdenticalClassMentionComparator.html]

```
001    package edu.uchsc.ccp.util.nlp.mention.comparison;
002    
003    import edu.uchsc.ccp.util.nlp.annotation.comparison.SpanComparator;
004    import edu.uchsc.ccp.util.nlp.mention.ClassMention;
005    import edu.uchsc.ccp.util.nlp.mention.Mention;
006    
007    /**
008     * This class implements a comparator which compares ClassMentions. ClassMentions must be identical.
009     * 
010     * @author williamb
011     * 
012     */
013    public class IdenticalClassMentionComparator extends IdenticalMentionComparator implements ClassMentionComparator {
014    
015        /**
016         * Compare two mentions. They must be ClassMentions or else an UnsupportedOperationException is thrown.
017         */
018        @Override
019        public int compare(Mention mention1, Mention mention2, SpanComparator spanComparator, int maximumComparisonDepth, int depth) {
020            if ((mention1 instanceof ClassMention) & (mention2 instanceof ClassMention)) {
021                return compare((ClassMention) mention1, (ClassMention) mention2, spanComparator, maximumComparisonDepth, depth);
022            } else {
023                throw new ClassCastException("Cannot support comparison between a ClassMention and a non-ClassMention");
024            }
025    
026        }
027    
028        /**
029         * 
030         * @param cm1
031         * @param cm2
032         * @param spanComparator
033         * @param maximumComparisonDepth
034         * @return
035         */
036        public int compare(ClassMention cm1, ClassMention cm2, SpanComparator spanComparator, int maximumComparisonDepth) {
037            return compare(cm1, cm2, spanComparator, maximumComparisonDepth, -1);
038        }
039        
040        /**
041         * This is a more specific compare() method for ClassMentions.
042         * 
043         * @param cm1
044         * @param cm2
045         * @param maximumDepth
046         *            specifies the depth in the mention hierarchy
047         * @param depth
048         * @param compareLinkedAnnotationSpans
049         *            toggles the comparison of the linked TextAnnotations. In some cases, the linked TextAnnotations may have already been compared, so
050         *            an infinite loop would be created by comparing them again.
051         * @return
052         * 
053         * TODO: we are assuming only a single text annotation per class mention
054         */
055        private int compare(ClassMention cm1, ClassMention cm2, SpanComparator spanComparator, int maximumComparisonDepth, int depth) {
056    
057            /* increment the depth level */
058            depth++;
059    
060            if (hasEquivalentMentionNames(cm1, cm2)) {
061                /*
062                 * the mention names are identical, so we need to now check the slots and complex slots, unless we have reached the maximum comparison
063                 * depth
064                 */
065                /* if we have reached the maximum comparison depth, then return now */
066                if (maximumComparisonDepth != -1 && depth >= maximumComparisonDepth) {
067                    return spanComparator.compare(cm1.getTextAnnotations().get(0).getSpans(), cm2.getTextAnnotations().get(0).getSpans());
068                } else {
069                    /* we have not yet reached the maximum depth, so we need to compare the slot mentions and complex slot mentions */
070                    boolean annotationSpansMatch = false;
071                    annotationSpansMatch = (spanComparator.compare(cm1.getTextAnnotations().get(0).getSpans(), cm2.getTextAnnotations().get(0)
072                            .getSpans()) == 0);
073    
074                    boolean slotMentionsMatch = equalSlotMentions(cm1.getSlotMentions(), cm2.getSlotMentions(), spanComparator,
075                            new IdenticalSlotMentionComparator(), maximumComparisonDepth, depth);
076                    boolean complexSlotMentionsMatch = equalComplexSlotMentions(cm1.getComplexSlotMentions(), cm2.getComplexSlotMentions(),
077                            spanComparator, new IdenticalComplexSlotMentionComparator(), maximumComparisonDepth, depth);
078    
079                    if (annotationSpansMatch & slotMentionsMatch & complexSlotMentionsMatch) {
080                        return 0;
081                    } else {
082                        return cm1.getSingleLineRepresentation().compareTo(cm2.getSingleLineRepresentation()) * MULTIPLIER;
083                    }
084                }
085            } else {
086                return cm1.getSingleLineRepresentation().compareTo(cm2.getSingleLineRepresentation()) * MULTIPLIER;
087            }
088        }
089    
090    }
```
